# Mechanical Properties and In Vitro Evaluation of Thermoplastic Polyurethane and Polylactic Acid Blend for Fabrication of 3D Filaments for Tracheal Tissue Engineering

**DOI:** 10.3390/polym13183087

**Published:** 2021-09-13

**Authors:** Asmak Abdul Samat, Zuratul Ain Abdul Hamid, Mariatti Jaafar, Badrul Hisham Yahaya

**Affiliations:** 1 Lung Stem Cell and Gene Therapy Group, Regenerative Medicine Cluster, Advanced Medical and Dental Institute (IPPT), Sains@Bertam, Universiti Sains Malaysia, Kepala Batas 13200, Malaysia; asmakas@iium.edu.my; 2 Fundamental Dental and Medical Sciences, Kulliyyah of Dentistry, International Islamic University Malaysia, Kuantan 25200, Malaysia; 3 School of Materials and Mineral Resources Engineering, Universiti Sains Malaysia, Nibong Tebal 14300, Malaysia; srzuratulain@usm.my (Z.A.A.H.); mariatti@usm.my (M.J.)

**Keywords:** thermoplastic polyurethane, polylactic acid, trachea scaffold, 3D filament

## Abstract

Surgical reconstruction of extensive tracheal lesions is challenging. It requires a mechanically stable, biocompatible, and nontoxic material that gradually degrades. One of the possible solutions for overcoming the limitations of tracheal transplantation is a three-dimensional (3D) printed tracheal scaffold made of polymers. Polymer blending is one of the methods used to produce material for a trachea scaffold with tailored characteristics. The purpose of this study is to evaluate the mechanical and in vitro properties of a thermoplastic polyurethane (TPU) and polylactic acid (PLA) blend as a potential material for 3D printed tracheal scaffolds. Both materials were melt-blended using a single screw extruder. The morphologies (as well as the mechanical and thermal characteristics) were determined via scanning electron microscopy (SEM), Fourier Transform Infrared (FTIR) spectroscopy, tensile test, and Differential Scanning calorimetry (DSC). The samples were also evaluated for their water absorption, in vitro biodegradability, and biocompatibility. It is demonstrated that, despite being not miscible, TPU and PLA are biocompatible, and their promising properties are suitable for future applications in tracheal tissue engineering.

## 1. Introduction

Tracheal injury can result from several conditions, including cancer, infection, trauma, or congenital anomalies. The conventional indication for therapy in severely injured tracheas of any aetiology is partial or full reconstruction, which necessitates the substitution of a graft or scaffold at the site of the lesion [1,2]. Unfortunately, even though there are a few treatment options available such as natural grafts or synthetic replacement, no optimal material has met the criteria. The limitations for natural grafts include the availability of donors and the unmatched size of donor grafts. According to the Organ Procurement & Transplantation Network, United States Department of Health and Services, the number of patients on the national transplant waiting list until July 2019 for all organ types has increased to more than 100,000. Out of this number, two-thirds are above the age of 50, while almost 2000 are below the age of eighteen and only one-third of the total numbers received organ transplantation [3]. In addition, the natural grafts derived from donors are challenged by the possibility of severe immune-rejection risks and complications caused by infection or disease from the donor-to-patient [4]. On the other hand, synthetic scaffolds are commonly associated with the biocompatibility of the scaffold material, inadequate mechanical properties, and biodegradability over time. In addition, other problems (such as availability for mass production with easy fabrication and the need for the size of the trachea should be custom-made to patients) persist [5,6,7].

Synthetic materials such as biodegradable polymers are gaining attention as materials in tissue engineering due to their broad processability window where the macro- and microstructures, mechanical properties, and degradation time can be easily manipulated and controlled. Scaffolds are fabricated and manipulated through various techniques to produce high precision, which suits the application. Some of the techniques used in the fabrication of tracheal scaffold using biodegradable materials that have been tested in animal models are electrospinning [8], thermally induced phase separation [9], and three dimensional (3D) printed technology (additive manufacturing) [10,11]. However, the choice of material and design for tracheal scaffold fabrication remains a challenge. To meet the selection criteria, many requirements must be fulfilled. For example, the scaffolds must create a suitable 3D niche for the cells to grow, proliferate, and differentiate, and should not elicit an immune reaction that can trigger a severe inflammatory response that might reduce healing or cause rejection by the body [12]. In vivo, the scaffold functions as a temporary framework that degrades over time which is eventually replaced by the body’s cells. Therefore, the degradation rate should match the rate at which the cells produce their cellular matrix, while the by-products released should be innocuous and eliminated safely through the body system [13]. Similarly, sufficient mechanical integrity of the implanted scaffold is required to allow for physiological functionality starting from implantation until the completion of the remodelling process.

Additive manufacturing (AM), also known as three-dimensional (3D) printing, has been used to fabricate tissue-engineered constructs. According to the ISO/ASTM standard, AM is defined as the “process of joining materials to make parts from 3D model data, usually layer upon layer” [14]. The AM is significantly different from traditional formative or subtractive manufacturing. It is the closest to ‘bottom up’ manufacturing, in which a structure can be built into its intended shape using a layer-by-layer technique. This layer-by-layer manufacturing technique enables unprecedented precision and control for constructing complex, composite, and hybrid structures. The four key components in AM include a digital model of the object, materials that are consolidated from the smallest possible form, a machine for laying materials, and a digital control system for the machine to lay the materials layer-by-layer to form a complex structure with customizable shape, size, and internal architecture [15,16,17]. The 3D fabrication of a tracheal scaffold has been reported in several preclinical studies using different types of polymeric materials such as polylactic acid [18] and polycaprolactone [19,20].

Thermoplastic polyurethane (TPU) is a polymeric material that can be manipulated, moulded, and produced through heating in various industrial processes. Polyurethane is composed of three materials; a diisocyanate, a chain extender and a macrodiol (or polyol) which are linked to form linear, segmented copolymers consisting of alternating hard and soft segments. The soft and flexible segment is derived from polyols such as polyester, while the rigid and hard segment is formed from the diisocyanate and chain extender [21]. TPU exhibits a broad range of mechanical properties across a wide range of temperatures due to the various ratios of soft to hard segments. As a result of its excellent physical properties and biocompatibility, it is widely used in biomedical applications, particularly in flexible uses such as blood vessels [22,23,24], catheters [25,26], and cartilage [27,28].

Polylactic acid (PLA) is a semi-crystalline polymer that belongs to the α-hydroxy acid family, derived from renewable sources such as corn, potatoes, sugarcane, and beets. It is classified as an aliphatic polyester because of the ester bonds that connect the monomer units, the lactic acids [29,30]. PLA and its copolymers have become one of the most attentively studied components in the biomedical field because of their excellent biological and mechanical properties, biodegradability and processability. Hence, it has wide applications such as medical implants, sutures [31], bone fixation screws [32], and drug delivery systems [33]. However, biodegradable PLA exhibits little to no elastic behaviour and is not favoured for applications requiring high flexibility or deformation in situ. Furthermore, the inherent hydrophobicity and slow degradability of PLA slightly impede its application in biological systems [34].

Blending two or more polymers is a common physical modification approach to enhance the existing properties of both materials to customize the desired properties for a particular application [34,35,36]. The blending technique has been utilized to overcome the limitations of the physical properties of polymers and has resulted in materials with novel properties such as shape memory and morphology that are not present in the parent polymers. These materials can be moulded into various structures, including films, porous scaffolds, fibres, filaments, and particles, depending on the intended application, with properties tuned for use in a variety of biomedical applications. Polymer blending facilitates the efficient and cost-effective modification or improvement of a polymer’s properties, thereby minimizing the significant costs and efforts associated with research and development of new polymers or copolymers. The blending techniques used are melt extrusion, foaming, electrospinning, and compression moulding [34,35].

Several biodegradable polymers that have been used to fabricate a tracheal scaffold are polylactic acid (PLA), polyglycolic acid (PGA), polylactide-co-glycolide acid (PLGA) [37,38,39,40], polypropylene [41,42], polyethylene terephthalate [43], high-density polyethylene (HDPE) [44], and polycaprolactone (PCL) [45,46,47,48]. Most of these polymers are used in combination with other synthetic or natural materials to enhance their properties.

Despite a large amount of research into various biodegradable polymers, clinical performance has yet to satisfy theoretical expectations. As a result, there is currently no clinically feasible solution for patients with long segmental airway problems. Therefore, an ideal synthetic scaffold that is biocompatible, timely degraded, and eliminated by the body system with appropriate and physical-mechanical qualities that can be easily replicated when needed (and is maybe individually custom-made to prevent prosthesis failure) is required. This study investigated the physical blending of two materials to obtain the optimum mechanical properties while retaining the material’s superior inherent properties. Additionally, it aimed to evaluate the physical and mechanical properties of a series of TPU and PLA blends, which will be used to produce filaments for 3D printing for tracheal tissue engineering. The TPU/PLA blended matrix, a combination of soft material TPU and rigid material PLA, is expected to act as an artificial ECM by possessing suitable mechanical strength and flexibility between the TPU and PLA.

## 2. Experimental

### 2.1. Materials

TPU Estane 58,311 NAT 028 (Brussel, Belgium); PLA NatureWorks, 2002D was purchased from NatureWorks LLC (Minnesota, MN, USA) with a specific gravity of 1.24 and a melt index of 5.0–7.0 g/10 min (2.16 kg loads at 210 °C).

### 2.2. Methods

#### 2.2.1. Fabrication of Polymer Blends’ Filaments via Melt Extrusion Technique

Prior to extrusion, the pellets of both polymers were dried in a 60 °C oven for 12 h. Then, the extrusion of fibres was performed using a Brabender (Duisburg, Germany) single screw extruder with a 1.75 mm die, operated according to the manufacturer’s instructions. A total weight of 100 g was used for each composition based on their weight percentage ratio (TPU: PLA) and coded as 100/0, 90/10, 80/20, 70/30, 60/40, and 0/100, respectively. Next, both materials were manually premixed via tumbling in a plastic zip-lock bag before melt-compounding. Once optimised, the temperature of the single screw extruder was set at 170° to 205 °C (±5 °C), the rotation speed was at 40 (±5) rpm, and the mixture was fed for melt compounding. Finally, the filaments were pelletised and hot-pressed into dumbbell shapes and 10 mm × 10 mm square samples, allowing for various characterisation methods. The TPU filament was produced using TPU pellets only in the same manner as other blends.

#### 2.2.2. Characterisation of Polymer Blends

##### Fourier Transform Infrared (FTIR)

FTIR spectra were obtained in a reflective absorbance mode on a Perkin Elmer spectrometer (Waltham, MA, USA) with a constant spectral resolution of 4 cm^−1^, in the range of 4000 to 550 cm^−1^, and after 16 scans. The reported spectra were analysed quantitatively using Perkin Elmer Software (Waltham, MA, USA) version 10.

##### Differential Scanning Calorimetry (DSC)

Thermal property analyses were carried out using a Differential Scanning Calorimetry (Mettler Toledo, Greifensee, Switzerland). In standard aluminium pans, about 10 mg of the samples were from room temperature to 250 °C at a rate of 20 °C/min and held isothermally for 5 min to exclude all previous thermal history. After cooling at 5 °C/min to −80 °C at a rate of 10 °C/min, samples were heated again at 20 °C/min to 250 °C. All of the experiments were conducted under a nitrogen atmosphere.

##### Mechanical Testing

The mechanical properties of all blends were conducted in tensile uniaxial mode using an Instron universal testing machine model 3366 (Norwood, MA, USA) at a crosshead speed of 5 mm/min according to ASTM D638. The samples analysed were 60 mm × 5 mm in size, with a thickness of around 1.0 mm. The slope of the straight-line stress-strain curve was used to calculate the Young’s Modulus (YM) of the polymer blends and the effects of tensile strength and percentage of elongation at break. The mean and standard deviation of five measures were used to calculate all results.

##### Scanning Electron Microscope (SEM)

SEM images were captured using a tabletop SEM (Hitachi, Tokyo, Japan). The cross-sectional surfaces were obtained from a tensile examination of a broken dumbbell. Before microscopy experiment, all specimens were sputtered with a thin layer of gold.

##### Water Absorption Study

The water absorption test was performed on 10 mm square samples according to ASTM D570. The samples were dried at 60 °C for 24 h to achieve a stable weight. The dried samples were weighed and placed in the test plate wells, prewetted with PBS solution before filling with 5 mL of PBS, pH 7.4 at 37 °C. The samples were incubated and tested at 8, 24, 48, and 72 h. The excess water was carefully removed with tissue paper, and the samples were re-weighed. Water absorption was calculated based on the amount of water absorbed according to Equation (1):

(1) $$Water\;absorption\;\left(\%\right)=\frac{Wwet-Wdry}{Wdry}\times 100$$

For each composition, three specimens were examined to achieve an average value.

##### In Vitro Degradation Study

PBS soaking tests were used to mimic the hydrolytic degradation activity of TPU/PLA blends and control scaffolds. The samples were weighed after drying overnight at 60 °C. Each sample was individually enclosed in a plastic container filled with a 1X PBS solution and incubated at 37 °C in an orbital shaker (Stuart S1500, Illinois City, IL, USA) at a shaking rate of 50 rpm. Every 7 days, PBS was refreshed, and the test lasted up to 7 months. The samples were rinsed three times with purified water before being dried overnight and weighed at each time point. Equation (2) was used to measure the weight loss of the materials.

(2) $$Degradation\;\left(\%\right)=\left(1-\frac{W_{n}}{W_{o}}\;\right)\times 100$$

*W*_0_ is the initial sample weight, and *W_n_* is the weight of the same sample after degradation for a time, *n*.

##### In Vitro Biocompatibility Assay

The blended samples were evaluated for biocompatibility and possible use as tissue engineering scaffolds in biomedical applications.

##### Pellet Sterilisation

Prior to in vitro testing, the TPU/PLA pellets were sterilised by being immersed in 70% ethanol (*v/v*) for 2 h, followed by rinsing three times with 1x PBS to eliminate all traces of ethanol. The pellets were then air-dried in a sterile atmosphere before being sterilised for 2 h with ultraviolet light. This phase ensured that any pollutants on the surface of the pellets were removed.

##### Pesto Blue Viability Assay

In order to be considered for biomedical applications, any material must not impose any toxicity to the surrounding tissues. The toxicity of the TPU and PLA was investigated in this study and was defined as a reduction in cell growth to less than 50% viability. The sterile pellets were immersed in complete α-MEM overnight before testing. BEAS-2-B (human bronchial epithelial cells) were purchased form American Type Culture Collection (ATCC, Manassas, VA, USA) were grown to confluence in complete α-MEM containing 10% FBS and 1% antibiotic–antimycotic (AA) solution at 37 °C in an incubator with 5% carbon dioxide (CO_2_). In 24-well plates, one pellet was placed in each well, followed by direct cell seeding on top of the pellet at a seeding number of 1 × 10^4^ cells per well, and the plates were incubated in a CO_2_ incubator for 3 days. As a positive control, a tissue culture plate with fresh α-MEM was used. The toxicity test was performed using Presto Blue Cell Viability Reagent on days 1, 2, and 3. The metabolic product of viable cells was released into the culture medium, reduced resazurin to resorufin, and changed the colour from blue to pinkish red. When reduction did not occur in a nonviable setting, the blue-coloured resazurin was preserved. An automatic ELISA reader was used to test the fluorescence of the samples in triplicate. Equation (3) was used to measure the cell toxicity percentage:

(3) $$\mathrm{Cell}\;\mathrm{toxicity}\;\left(\%\right)=\frac{Fluorescence_{treatment}-Fluorescence_{control\;}}{Fluorescence_{control}}\times 100$$

##### Statistical Analysis

The mean and standard deviation of all the results were calculated (SD). The one-way analysis of variance (ANOVA) was used for normally distributed data, while for non-normally distributed data, the nonparametric test was used. The Tukey’s test was then used to assess the data’s particular differences, with *p* < 0.05 indicating statistical significance. The data were analysed using GraphPad Software (Prism 9.0, GraphPad Software, La Jolla, CA, USA).

## 3. Results and Discussion

Apart from being biocompatible and biodegradable, as the foundation of the tracheal structure, the material of the scaffold should have adequate mechanical strength and flexibility to enable physiological function during breathing. The rigid components of the cartilage retain the trachea lumen open and prevent its collapse under negative air pressure, preventing airflow limitations [49]. The purpose of this study is to determine the physical and mechanical properties, as well as the absorption, in vitro degradation, and biocompatibility, of a series of TPU and PLA blends that will be produced as filaments and 3D printed as a potential material for tracheal replacement.

### 3.1. Fabrication of Filament and Identification of the Materials

**Figure 1:**
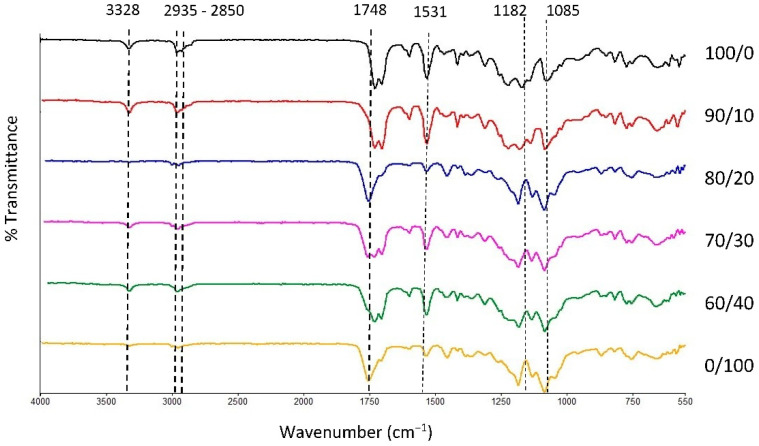
FTIR spectra of TPU, PLA, and TPU/PLA blends.

The physical blending of the material through the melt extrusion technique of TPU and PLA was chosen in this study to produce filaments that will be fabricated as tracheal scaffold using a 3D printing technique. Both materials were mixed based on their weight percentage and characterised accordingly. FTIR transmission spectra of the samples are presented in Figure 1 with main characteristic bands of pure TPU appeared at 3328 cm^−1^, 2935 cm^−1^, and 2850 cm^−1^ which correspond to stretching of -NH- in urethane and asymmetric and symmetric vibrations in -CH2- respectively [50,51,52,53]. In addition, 1700, 1531 and 1314 cm^−1^ bands were associated with bending and stretching of amide I, II, and III bonds. The intensity of characteristic bands in TPU reduces as the PLA contents increases and vice versa. The characteristic bands of PLA were seen at 1750 cm^−1^, 1456 cm^−1^, and 1180 cm^−1,^ corresponding to asymmetric vibration of -C=O and the asymmetric and symmetric stretching of -C-O-C bonds, respectively. The characteristic bands and their activities are summarised in Table 1. Overall, the spectra revealed no new chemical bonds, suggesting that both PLA and TPU were successfully compounded during melt blending [54,55]. Physical blending does not alter the chemical characteristic of the constituents of the polymers [34]. Hence, the properties of the blend can be conveniently customized using different compositions of the polymer to suit its application.

### 3.2. Morphology and Miscibility of the Blends

**Figure 2:**
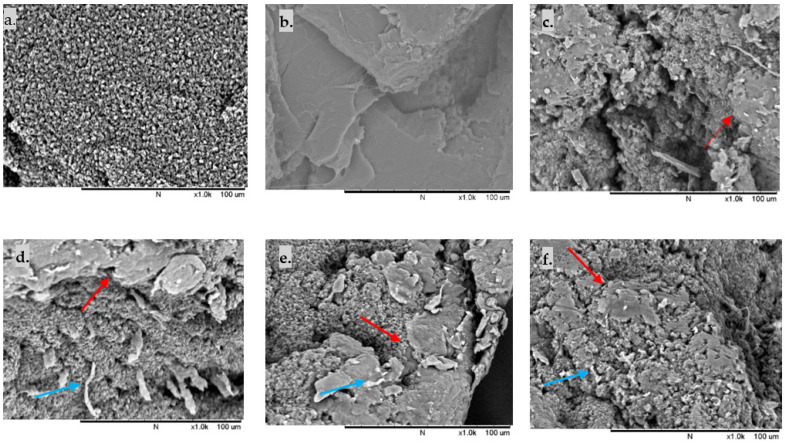
Cross-section SEM images of reactively extruded films: (**a**) 100/0, (**b**) 0/100. Homogeneous matrices are present in pure TPU and pure PLA (**c**) 90/10, (**d**) 80/20, (**e**) 70/30, (**f**) 60/40. Some fibrous TPU is present in the polymer blends, with PLA domains are dispersed in TPU matrices in all blends. Red arrows show PLA particles in the TPU matrix, while blue arrows indicate the fibrous TPU of the fractured surface. Scale bar of 100 μm.

The SEM images of the fractured surface TPU/PLA dumbbell films were analysed to study the phase morphology of the samples. In contrast, the DSC findings were used to determine the thermal properties and miscibility of the samples. As shown in Figure 2, the fractured surface morphology of pure TPU and pure PLA reveals the homogeneous distribution of fibrous TPU and continuous matrix of PLA, respectively. In contrast, in blended compositions, the smooth-edged PLA domains are distributed in the fibrous TPU matrix, resulting in a notably heterogeneous two-phase structure. Both 90/10 and 80/20 samples displayed more fibrous TPU than 70/30 and 60/40 samples, reflecting the flexibility of the blends. However, several separated phase domains with minute debonded holes were also observed, indicating a weak interfacial contact between the TPU matrix and the PLA domain. As the PLA ratio increased, larger and more PLA spheres were detected in the TPU matrix, although they were dispersed equally. All blended polymers exhibit phase separation, suggesting that TPU and PLA are immiscible.

**Figure 3:**
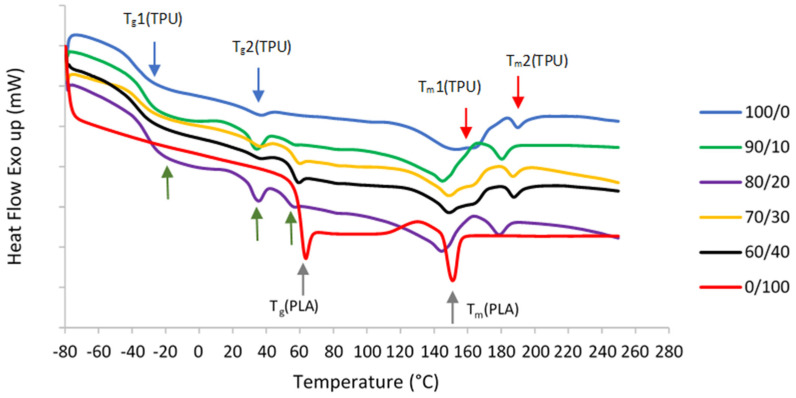
DSC secondary melting curves of pure TPU and PLA, and TPU/PLA blends. The blue arrows show the first and second T_g_ values of pure TPU, while red arrows indicate both T_m_ values of pure TPU. Grey arrows show the T_g_ and T_m_ values of pure PLA. Finally, the green arrows depict all three T_g_ values of TPU/PLA blends.

TPU is composed of diisocyanate hard segments and polyester macroglycol soft segments, while PLA is an aliphatic polyester. Due to their structures, two glass transition temperature (T_g_) and melting temperature (T_m_) values were observed in pure TPU, which correspond to hard and soft segments as shown in DSC curves in Figure 3. On the other hand, only one T_g_ and T_m_ were seen in pure PLA.

The immiscibility of the blends is further demonstrated by DSC curves and summarized data as shown in Table 2. The T_g_ values can determine the extent of blend miscibility, partial miscibility, or total immiscibility as a function of the polymer blend composition. Normal T_g_ values of the TPU and PLA ranges from around −18 °C to −47 °C and 61 °C to 67 °C, respectively [56,57]. Miscible polymers typically have a single T_g_, whereas immiscible blends transition temperatures shift toward each other to a degree depending on the mutual miscibility of the phases [58,59,60,61]. In the event of a completely immiscible blend system, the blend components may remain their original T_g_ values, regardless of the blend composition [34]. In this study, the appearance of three distinct T_g_ values in 90/10, 80/20, 70/30, and 60/40 samples suggest that TPU and PLA are immiscible. Two T_g_ values correspond to the TPU structure, whereas another T_g_ value comes from the PLA. The immiscibility was demonstrated by considerable macroscopic phase separation in the blends in SEM. Moreover, all mixed samples that confirm the immiscibility also show two different melting temperatures (T_m_). This result is in line with [51,61,62,63]. In immiscible blends, the properties of the component polymers integrate in such a way that the blend morphology is a direct representation of the component morphology. As a result, blend morphology is regarded as a good indicator of blend miscibility.

### 3.3. Mechanical Properties

**Figure 4:**
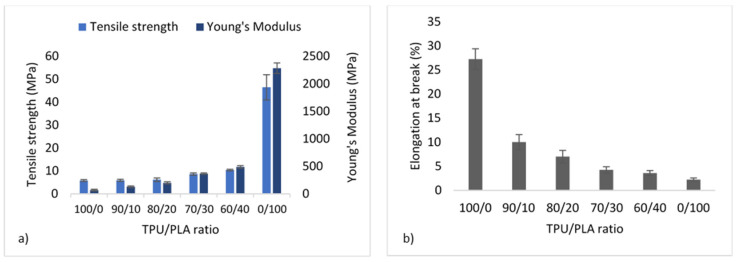
Mechanical properties of TPU/PLA blends showing (**a**) tensile strength and Young’s modulus in MPA, and (**b**) percentage of elongation at break.

It is well recognised that the morphology of the materials greatly influences the mechanical properties of blends, and the properties of the final product can be achieved by adjusting the morphology. The tensile strength and YM measurement were derived from the stress–strain curve of the tensile test ASTM D638. Figure 4 depicts the mechanical properties of the TPU/PLA blends. Pure PLA has the highest tensile strength and YM at 46.48 ± 5.51 MPa and 2282.80 ± 95.60 MPa, preceded by TPU blends with higher PLA contents. This means that the higher the PLA concentration, the stiffer the blend. On the other hand, pure PLA demonstrated the least amount of flexibility because it cannot elongate by more than 5% due to its inelastic nature. In comparison, pure TPU showed the highest flexibility (up to more than 25%) without fracture, as shown in Figure 4b. The flexibility decreases with decreasing TPU concentration, with the 60/40 blend exhibiting the least flexibility of all blends. The morphology of TPU and PLA blends appeared to be related to their mechanical properties, with stretched fibrous TPU can be seen in fractured surfaces images of the 90/10 and 80/20 samples reflecting the blends’ flexibility.

**Figure 5:**
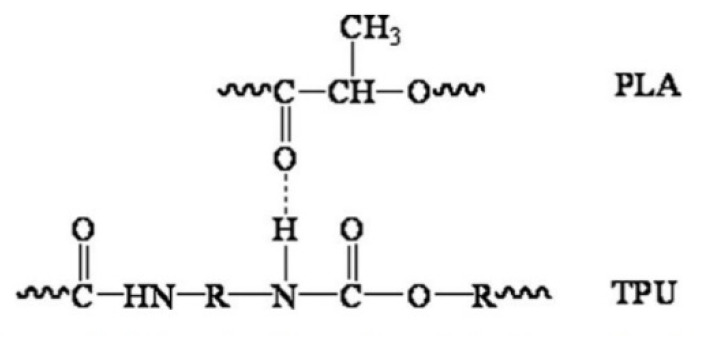
Schematic diagram of hydrogen bonding between the molecules of PLA and TPU. Reproduced with permission from Feng and Ye, Journal of Applied Polymer Science; reprinted with permission from ref [69], Copyright 2011 John Wiley and Sons.

As a substitute for the cartilage wall, the scaffold is expected to possess adequate strength to keep the airway open and resist collapse. At the same time, it maintains the flexibility to allow flexion/bending despite intrathoracic pressure differences during breathing cycles [63,64]. TPU is known to have flexibility and is widely used in soft tissue engineering [23,24,65,66]. The present study revealed that the tensile strength and Youngs’ modulus of TPU were proportionately increased when PLA was added. In contrast, the percent elongation reduces accordingly. The results obtained were similar to those reported by Lis–Bartos et al. (2018) [67] and Mi et al., (2013) [51]. The overall physicomechanical behaviour of immiscible systems is critically dependent on two demanding structural parameters. First, an appropriate interfacial tension leads to a phase size that is small enough to allow the material to be considered macroscopically homogeneous. Secondly, an interphase adhesion is strong enough to assimilate stresses and strains without disrupting the established morphology [68]. Even though the blend was not miscible, it is noted that they considered having good compatibility with each other. This is due to the composition of diisocyanate hard segments and polyester macroglycol soft segments of the TPU and aliphatic polyester in PLA, which forms hydrogen bonding between the molecules of the blend, as shown in Figure 5.

The blend is suitable for the fabrication of a tissue-engineered scaffold since the mechanical properties are within the normal human tracheal range: tensile stiffness 1–15 MPa [70] and YM 12.2–20.5 MPa in young and old humans; ~16 MPa in both human male and female [64].

The purpose of combining two polymers is to produce a material with improved physical properties over the parent polymers. The degree of modification is relative to the quantity of each composition of the polymer. The morphology of the blend and the dispersion of the phases have a considerable effect on the thermomechanical characteristics. For example, TPU with a lower tensile strength is combined with PLA to increase the tensile strength. Despite the modest reduction in elongation at break, the final material can be adjusted by varying the polymer composition to attain the optimal combination of thermal and mechanical properties for specific applications.

### 3.4. Water Absorption and In Vitro Degradation Rate

An ideal implant material should possess optimal absorption and biodegradable properties similar to the regenerative process of the native tissue [71]. This fills up surrounding tissues and provides a biocompatible framework that allows cells, blood vessels, and newly created tissues to develop and maintain an extracellular matrix [72].

The swelling behaviour of a material (or its ability to absorb water) is an important factor to consider when constructing a scaffold. Although excessive water absorption damages the scaffold’s morphology, its absence causes inadequate absorption, inhibiting cell growth in vivo [54]. The absorption rate was determined by immersing the samples in PBS solution. Pure PLA showed the highest absorption rate in PBS solution, which was approximately 5% in the first hour, decreased significantly after 8 h, and remained stable until 48 h. On the other hand, other compositions exhibited nearly equal absorption, varying from 1 to 2 percent, but decreased steadily and remained nearly stable until 48 h. No significant differences were found in any of the compositions. The material’s absorption property indicates the scaffold’s ability to bind to the surrounding metabolites and promotes the transportation of nutrients and cell integration throughout the scaffold [73].

**Figure 6:**
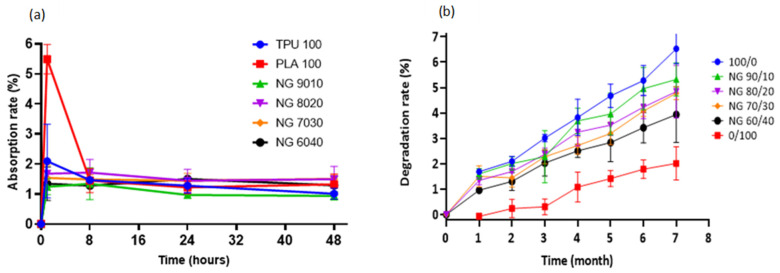
(**a**) Water absorption rate of TPU, PLA, and the blends, (**b**) In vitro degradation study of the samples. Pure TPU shows the fastest degradation rate, while the slowest is that of pure PLA.

One of the primary goals of biodegradable tissue-engineered scaffolds is to provide a mechanical structure or framework to support the extracellular matrix (ECM). At the same time, it degrades evenly/slowly via hydrolytic degradation, allowing the surrounding tissue to recover the supporting function of the scaffold [74]. The monomeric components of the polymers are innocuously eliminated through the body system [75]. In the degradation study over 7 months, all of the samples showed a gradual loss with pure TPU (100/0) exhibiting the fastest degradation rate, followed by 90/10, 80/20, 70/30, 60/40, and pure PLA (0/100). The higher the TPU concentration, the faster rate of degradation. The degradation percentage up to 7 months was less than 6%, suggesting that the blended material is long-term stable. In contrast, the degradation of PLA is slower, which started only after 1 month of incubation and gradually degraded at a very slow rate. Up until 7 months, the degradation percentage was approximately 2% and was the lowest among other blends. Figure 6 shows both the rate of absorption and degradation results.

The biodegradation of polymeric biomaterials mainly depends on hydrolytic degradation of polymer chains. For example, degradation of polyurethane occurs when water molecules infiltrate the polymer network, triggering hydrolysis of the polyurethane chains, including the chemical dissolution of ester and amide bonds [76]. Similarly, the hydrolytic degradation of PLA started by the breaking of the ester link of the polymeric chain [77]. Hydrolytic degradation has been described as belonging to two types: surface erosion and bulk erosion. Surface erosion occurs exclusively at the polymer–water interface, while bulk erosion occurs uniformly throughout the polymer [76,77]. However, it is critical to emphasise that this degradation investigation was conducted on nonporous film specimens without plasma, biomolecules, or cells. The rate of degradation is important, as it is controlled by various other factors in vivo, which significantly enhances the degradation of polymers.

Erosion and bulk degradation are the two possible mechanisms through which polymers degrade. Crystallinity and chain orientation of polymers are particularly essential in degradation processes. The degradation process begins with water diffusion into the amorphous regions, followed by random hydrolytic scission of the ester bonds. After degrading the amorphous sections, the hydrolytic attack continues to the crystalline structures. Due to the loose packing of the molecules in the amorphous area of a polymer, they are more susceptible to attack by reactive species or solvents than the molecules in the crystalline region [78,79,80]. TPU’s amorphous structure is the possible cause that results in a faster degradation rate than semicrystalline PLA, as seen by its lower glass transition temperature (T_g_).

### 3.5. In Vitro Biocompatibility

**Figure 7:**
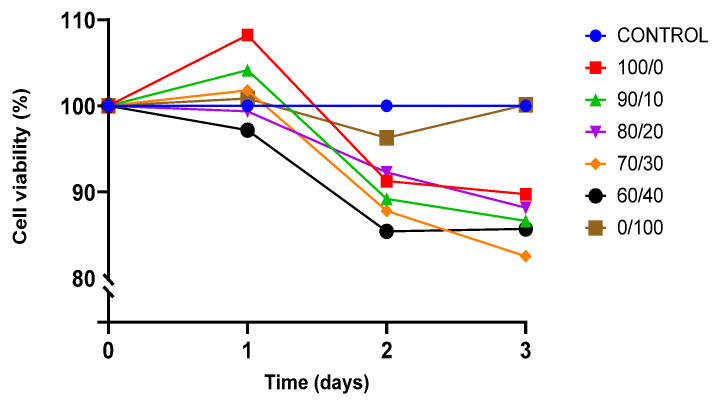
Presto Blue viability assay towards BEAS-2B cells.

The viability of cell culture is shown by the proportion of viable cells in a population. A decrease in the percentage of viable cells below 50% of total cell growth is described as the toxicity of the material towards the cells. BEAS-2B was cultured for three days on sterilised TPU, PLA, and TPU/PLA scaffolds to test their biocompatibility and toxicity of the material, and the result is shown in Figure 7.

Both pure TPU and PLA indicated biocompatibility towards BEAS-2B cells. After 24 h of incubation, cell proliferation was noted, exhibiting viability of cells was more than 100% in both pure materials. The result showed that the viability of BEAS-2B was greater than 80% in all compositions up until day 3, indicating that none of the compositions were toxic to the cells. Pure PLA outperformed pure TPU and other blends in terms of viability. TPU has been proven to be biocompatible and suitable for regenerative medicine, either alone or in conjunction with other polymers. Therefore, this result was expected [17,81,82]. This study is also consistent with the findings by Harynska and colleagues (2018) [83]. Similarly, the U.S. Food and Drug Administration (FDA) has authorised PLA as a biodegradable and biocompatible polymer for application in the human body due to its absorbability and nontoxicity [33,35,73,84].

## 4. Conclusions

Due to the shortage of organs and tissues for organ transplantation, synthetic materials are some of the treatment alternatives for trachea replacement, which necessitates developing an ideal material with desirable properties. Apart from biocompatibility and nontoxicity in the body system, mechanical properties are some of the important factors that must be considered due to the anatomy of the trachea in the body. TPU and PLA were selected for this study because of their inherent properties and wide use in biomedical applications. However, the properties of these materials limit their application. Polymer blending is an appealing and cost-effective method for developing new material with improved properties by combining physically existing polymers instead of synthesizing entirely new polymeric materials. This study investigated the mechanical properties, water absorption, biodegradability, and biocompatibility of melt blended TPU and PLA polymers as a material of choice in 3D printed tracheal tissue engineering. It was demonstrated that both materials were successfully compounded. Even though all blend compositions were not miscible, their morphology and mechanical properties showed improvement for the proposed use. Furthermore, the blended material is biocompatible and has an appropriate absorption and degradation rate, making it viable for use as a filament material in additive manufacturing for medical purposes. Therefore, the polymer blending of TPU and PLA can be a cost-effective strategy to improve the properties of TPU and PLA and produce filaments for 3D printing.

## Figures and Tables

**Table 1:** Characteristic bands and corresponding activities of TPU and PLA.

Characteristic Band	Activity	Material
3328	Stretching -NH- in urethane	TPU
2935	Asymmetric vibration in -CH2-	TPU
2850	Symmetric vibration in -CH2-	TPU
1748	-C=O stretching (amide I)	TPU/PLA
1531	N-H bending vibration (amide II)	TPU
1314	C-N (amide III)	TPU
1182	asymmetric stretching of -C-O-C-	PLA
1085	symmetric stretching of -C-O-C-	PLA

**Table 2:** Differential scanning calorimetry data for TPU, PLA, and blends.

Sample	T_g TPU_	T_g PLA_	T_m_1	T_m_2
100/0	−26.98	-	153.88	189.90
0/100	-	63.16	150.74	-
90/10	−25.12	58.21	145.18	180.20
80/20	−19.70	55.87	144.11	178.44
70/30	−27.62	59.24	148.17	186.84
60/40	−28.00	58.56	148.15	187.15

T_g_ = glass transition temperature, T_m_ = melting temperature.

## Data Availability

Not applicable.
